# Dietary acid load on the Mediterranean and a vegan diet: a secondary analysis of a randomized, cross-over trial

**DOI:** 10.3389/fnut.2025.1634215

**Published:** 2025-06-25

**Authors:** Hana Kahleova, Cristina Maracine, Joseph Himmelfarb, Arathi Jayaraman, Tatiana Znayenko-Miller, Richard Holubkov, Neal D. Barnard

**Affiliations:** ^1^Physicians Committee for Responsible Medicine, Washington, DC, United States; ^2^School of Medicine, University of Utah, Salt Lake City, UT, United States; ^3^George Washington University School of Medicine and Health Sciences, Washington, DC, United States

**Keywords:** dietary acid load, Mediterranean, nutrition, plant-based, vegan

## Abstract

**Background:**

Evidence suggests that changes in dietary acid load may influence body weight, and the purpose of this secondary analysis was to assess its role in the context of the Mediterranean and a vegan diet in overweight adults.

**Methods:**

In this randomized cross-over trial, 62 overweight adults were randomized to a Mediterranean or a low-fat vegan diet for 16-weeks, separated by a 4-week washout. Change in body weight was the primary outcome. Three-day dietary records were analyzed, and Potential Renal Acid Load (PRAL) and Net Endogenous Acid Production (NEAP) were calculated as markers of dietary acid load, and their relationship was tested with changes in body weight.

**Results:**

Compared with no change on the Mediterranean diet, PRAL and NEAP significantly decreased on the vegan diet; effect sizes: −25.8 (95% CI −34.1 to −17.5); *p* < 0.001; and −27.1 (95% CI −35.4 to −18.7); *p* < 0.001, respectively. Across both diets, changes in PRAL and NEAP were positively associated with changes in body weight in the first 16 weeks of the study: *r* = +0.34; *p* = 0.009; and r = +0.39; *p* = 0.002, respectively, as well as in the second 16 weeks: *r* = +0.59; *p* < 0.001, and *r* = +0.61; *p* < 0.001, respectively.

**Conclusion:**

These findings suggest that, compared with the Mediterranean diet, dietary acid load decreased significantly on the low-fat vegan diet and was associated with weight loss. The alkalizing effect of a vegan diet may be an independent mechanism by which a vegan diet promotes weight loss.

**Clinical trial registration:**

https://clinicaltrials.gov/, identifier NCT03698955.

## Introduction

A high dietary acid load has been associated with chronic low-grade metabolic acidosis, inflammation, and obesity (1). Moreover, studies found a dose–response relationship between dietary acid load, incidence of metabolic syndrome (2), and risk of cardiovascular disease (3).

Dietary acid load is commonly estimated by two scores. Potential Renal Acid Load (PRAL) estimates the effect of foods on the pH balance, based on five nutrient values (protein, phosphorus, potassium, magnesium, and calcium) (4). The second score, the Net Endogenous Acid Production (NEAP), further adjusts for the participant’s height and weight (5).

Dietary phosphorus and protein found in meat, fish, eggs, cheese, and certain grains release acid precursors (4, 6). In contrast, most fruits and vegetables have an alkalizing effect (4, 6). Alkaline diets have been associated with several health benefits, including weight loss, improved insulin sensitivity, and lower blood pressure (1).

Plant-based diets are typically associated with lower net acid excretion and higher urinary pH levels, compared with diets high in animal products (7). A cross-sectional study of three dietary patterns (flexitarian, lacto-ovo-vegetarian, and vegan) found a stepwise reduction in dietary acid load. The vegan diet displayed the highest alkalizing potential, followed by the lacto-ovo vegetarian and the flexitarian diets (8). A previous randomized clinical trial demonstrated that after adopting an *ad libitum* vegan diet, the reduction in dietary acid load was associated with weight loss in overweight adults (9).

Mediterranean and vegan diets have long been studied for their beneficial health effects, but the extent to which these dietary patterns affect dietary acid load still needs to be explored. This secondary analysis of a randomized crossover trial, which compared a Mediterranean and low-fat vegan diet head-to-head in overweight adults (10), assessed changes in dietary acid load and its association with changes in body weight.

## Methods

The overall study methods have been described earlier (10). Briefly: This randomized, cross-over trial was conducted as a single-center study in Washington, DC. The protocol was approved by the Chesapeake Institutional Review Board. All participants provided written informed consent.

Overweight participants were randomly assigned into 2 groups. The first one started with a Mediterranean diet, and the second one with a low-fat vegan diet. After the first 16 weeks, followed by a 4-week wash-out period, the participants switched to the opposite diet (Supplementary Figure 1). All outcomes were measured at weeks 0, 16, 20, and 36.

The Mediterranean diet followed the PREDIMED protocol (11), which includes fruits, vegetables, legumes, nuts or seeds, fish or shellfish, and favors lean white meats over red meats. Participants were asked to use 50 g of extra virgin olive oil every day. The low-fat vegan diet consisted of vegetables, grains, fruits, and legumes.

The participants completed a 3-day food diary at week 0, 16, 20, and 36. These were then analyzed by a registered dietitian certified in the Nutrition Data System for Research (12). Dietary adherence was checked weekly.

The primary outcome of this analysis was dietary acid load, and its relationship with changes in body weight. The dietary acid load was calculated as follows (13, 14):


$$\begin{array}{l} \text{PRAL}\;(\mathrm{mEq}/\mathrm{day})=(0.49\times \text{protein}\;[g/\mathrm{day}])\\ +(0.037\times \text{phosphorus}\;[\mathrm{mg}/\mathrm{day}])\\ –(0.021\times \text{potassium}\;[\mathrm{mg}/\mathrm{day}])–(0.026\times \text{magnesium}\;[\mathrm{mg}/\mathrm{day}])\\ –(0.013\times \text{calcium}\;[\mathrm{mg}/\mathrm{day}]) \end{array}$$

NEAP(mEq/day)={(0.49×protein[g/day])+(0.037×phosphorus[mg/day])–(0.021×potassium[mg/day])–(0.026×magnesium[mg/day])–(0.013×calcium[mg/day])}+{(0.007184)×(height[cm]^0.725)×(weight[kg]^0.425)×(41/1.73)}

This method of calculation was previously validated against urinary renal net acid excretion and shown to reliably estimate the acid load from diet composition (5). Physical activity was assessed by the International Physical Activity Questionnaire (IPAQ) (15). Body composition was measured by dual energy x-ray absorptometry (Lunar iDXA, GE Healthcare; Madison, WI) with Encore^®^ 2005 v.9.15.010 software.

### Statistical analysis

The statistical analysis was performed in all participants with complete data across all timepoints by a statistician blinded to dietary interventions. Treatment effect was quantified by comparing changes from baseline (from week 0 to week 16, and from week 20 to week 36), within study participants while on Mediterranean versus vegan diet, using paired t-tests. The carryover effect was then assessed by comparing treatment effects by the first diet that each participant received using two-sample t-tests. Spearman correlations were used to assess the relationship between changes in body weight and changes in PRAL and NEAP in the first and second 16 weeks of the study, across the study diets. After Bonferroni correction, *p*-values less than 0.006 (0.05/8) were considered significant. All results are presented as means with 95% confidence intervals (CI).

## Results

Of 506 people screened by telephone, 62 met participation criteria and were randomly assigned to start with the Mediterranean (*n* = 32) or the vegan (*n* = 30) diet (Supplementary Figure 1). Dietary adherence was consistently high throughout the study in both groups.

Compared with no change on the Mediterranean diet, PRAL and NEAP significantly decreased on the vegan diet; effect sizes: -25.8 (95% CI -34.1 to −17.5); *p* < 0.001; and −27.1 (95% CI −35.4 to −18.7); *p* < 0.001, respectively (Table 1). There was no significant carryover effect in PRAL or NEAP (Supplementary Table 1).

**Table 1:** Changes in dietary acid load during the study comparing a Mediterranean and low-fat vegan diet.

Category	Mediterranean baseline	Mediterranean final	ΔMediterranean	Vegan baseline	Vegan final	ΔVegan	Treatment effect	*P*-value
Dietary acid load (mEq/day)								
PRAL	+1.2 (−4.0 to +6.4)	−1.6 (−7.4 to +4.2)	−2.8 (−9.8 to +4.2)	9.3 (4.4 to 14.3)	−19.3 (−23.8 to −14.7)	−28.6 (−33.2 to −23.9)***	−25.8 (−34.1 to −17.5)	<0.001
NEAP	49.8 (44.4–55.3)	47.0 (40.7–53.3)	−2.8 (−9.9 to +4.2)	58.5 (53.1–63.9)	28.6 (23.7–33.5)	−29.9 (−34.6 to −25.2)***	−27.1 (−35.4 to −18.7)	<0.001

Data are means and estimated treatment effects with 95% confidence intervals. *p* values for treatment effect are from a two-sample *t*-test comparing mean changes between participants in each treatment arm. *p < 0.05, **p < 0.01, and ***p < 0.001 for within-group changes from baseline assessed by paired comparison *t*-tests.

Body weight was reduced by 6.0 kg on the vegan diet, compared with no change on the Mediterranean diet (treatment effect −6.0 kg [95% CI −7.5 to −4.5]; *p* < 0.001). Most of the weight loss was attributable to a reduction in body fat (treatment effect −3.5 kg [95% CI −4.7 to −2.2]; *p* < 0.001).

In the first 16 weeks of the study, across both diets, changes in PRAL and NEAP were positively associated with changes in body weight: *r* = +0.34; *p* = 0.009; and *r* = +0.39; *p* = 0.002, respectively. That is, reductions in dietary acid load were directly associated with reductions in body weight. The associations were attenuated after adjusting for changes in energy intake: *r* = +0.25; *p* = 0.06; and *r* = +0.31; *p* = 0.02, respectively. In the second 16 weeks, across both diets, changes in PRAL and NEAP were also positively associated with changes in body weight: *r* = +0.59; *p* < 0.001, and *r* = +0.61; *p* < 0.001, respectively. These associations remained significant even after adjustment for changes in energy intake: *r* = +0.48; *p* < 0.001, and *r* = +0.51; *p* < 0.001, respectively (Table 2).

**Table 2:** Spearman correlations between changes in body weight and changes in dietary acid load in the first and second 16 weeks of the study, across the study diets.

Category	Δ Body weight, first 16 weeks	*Δ* Body weight, second 16 weeks
PRAL	*r* = +0.34; *p* = 0.009	*r* = +0.59; *p* < 0.001
PRAL, adj. for energy intake	*r* = +0.25; *p* = 0.06	*r* = +0.48; *p* < 0.001
NEAP	*r* = +0.39; *p* = 0.002	*r* = +0.61; *p* < 0.001
NEAP, adj. for energy intake	*r* = +0.31; *p* = 0.02	*r* = +0.51; *p* < 0.001

After Bonferroni correction, *p*-values less than 0.006 (0.05/8) are considered significant.

## Discussion

This randomized cross-over trial demonstrated that, compared with the Mediterranean diet, dietary acid load significantly decreased on a vegan diet and was associated with weight loss, independent of energy intake.

This is consistent with previous observational studies, showing that dietary acid load is an independent risk factor for diabetes (16). Dietary acid load has been shown to affect body weight. Consumption of animal proteins and sodium chloride increases dietary acid load and induces metabolic acidosis by the accrual of non-metabolizable anions, primarily sulfate and chloride (7). A potential mechanism by which metabolic acidosis may contribute to weight gain is by increasing the secretion of glucocorticoids (7), which play a critical role in the maintenance of acid–base balance, adiposity, and insulin sensitivity (17). In contrast, fruits and vegetables are rich in potassium salts of metabolizable anions, which deplete hydrogen ions and therefore have an alkalizing effect (7). Furthermore, plant proteins are often high in glutamate, an amino acid that dissipates hydrogen ions during metabolism (7). Therefore, plant foods may significantly reduce the dietary acid load.

Several observational studies have found that, compared with omnivorous diets, plant-based diets tend to have a lower dietary acid load. Data from the National Health and Nutrition Examination Survey (2007–2010) showed that foods consumed by vegetarians had a significantly lower dietary acid load than that of nonvegetarians (18).

Similarly, observational data showed a lower dietary acid load on the Mediterranean diet, compared with a conventional dietary pattern. In a cross-sectional survey of 346 students at the University of the Peloponnese in Greece, the dietary acid load was negatively associated with the Mediterranean diet adherence score (19). This study suggests that greater adherence to Mediterranean-style eating may reduce the dietary acid load. A different observational study of 459 people in Italy found that dietary acid load, but not Mediterranean diet adherence, was associated with markers of cardiometabolic health (20).

Randomized controlled trials have found significant differences in dietary acid load between vegan and omnivorous diets. A 2021 randomized controlled trial in Germany showed that, compared with a meat-rich diet, a vegan diet significantly lowered dietary acid load (21). Another randomized trial of 244 overweight adults in the United States found a significant reduction in dietary acid load on a vegan diet, which was associated with weight loss, improved body composition, and insulin sensitivity, independent of energy intake (9). Randomized clinical trials exploring the effect of the Mediterranean diet on dietary acid load are missing, highlighting the importance of this cross-over trial.

### Strengths and limitations

The strengths of the current trial include a randomized, cross-over design. The duration of the study was sufficiently long enough for adaptation to both diets. Considering the participants were residing at home and preparing their own meals or eating at restaurants, these results are applicable outside the research setting, in free-living conditions. The study also has limitations. The food consumption and dietary acid load calculations were based on self-reported diet records. Attrition is another common limitation of nutrition studies. In this trial, 84% of the participants completed the whole study. Finally, the participants were volunteers and may not represent the general population, although they may represent the population of individuals seeking to lose weight.

## Conclusion

In conclusion, compared with a Mediterranean diet, dietary acid load decreased significantly on the low-fat vegan diet, which was associated with weight loss, independent of changes in energy intake. The alkalizing effect of a vegan diet may be an independent mechanism by which a vegan diet promotes weight loss.

## Data Availability

The raw data supporting the conclusions of this article will be made available by the authors, without undue reservation.
