# Supplementary material for: Dietary acid load on the Mediterranean and a vegan diet: a secondary analysis of a randomized, cross-over trial
Source: Front Nutr. 2025 Jun 25;12:1634215. doi: 10.3389/fnut.2025.1634215 (PMC12237638; doi:10.3389/fnut.2025.1634215)
Supplement: Supplementary file 1 [file Table_1.DOCX]

| Category | ∆Mediterranean (Participants with First Diet Mediterranean) | ∆Vegan  (Participants with First Diet Mediterranean) | Treatment Effect: Participants whose First Diet was Mediterranean | P-value for Participants whose First Diet was Mediterranean | ∆Mediterranean (Participants with First Diet Vegan) | ∆Vegan  (Participants with First Diet Vegan) | Treatment Effect: Participants whose First Diet was Vegan | P-value for Participants whose First Diet was Vegan | P-value: test for carryover effect |
| --- | --- | --- | --- | --- | --- | --- | --- | --- | --- |
| **Dietary acid load (mEq/day)** | | | | | | | | | |
| PRAL | -7.6 (-19.4 to +4.2) | -30.9 (-37.1 to -24.8)*** | -23.4 (-36.1 to -10.6) | <0.001 | +1.6 (-6.7 to +9.9) | -26.4 (-33.6 to -19.2)*** | -28.0 (-39.6 to -16.4) | <0.001 | 0.58 |
| NEAP | -7.9 (-19.8 to +4.0) | -31.9 (-38.1 to -25.6)*** | -24.0 (-36.7 to -11.2) | <0.001 | +1.9 (-6.5 to +10.2) | -28.1 (-35.4 to -20.8)*** | -30.0 (-41.6 to -18.3) | <0.001 | 0.48 |

**Suppl. Table 1. Changes in dietary acid load and estimated treatment effects for the first and the second period of the study, comparing a Mediterranean and a low-fat vegan diet.** Data are means and estimated treatment effects with 95% confidence intervals. P values for treatment effect are from a two-sample *t*-test comparing mean changes between participants in each treatment arm within each period. The P-value for a carryover effect is for significance of an interaction between initial treatment assignment and period, in a mixed model of treatment effect as described in Methods. *p<0.05, ** p<0.01 and *** p<0.001 for within-group changes from baseline assessed by paired comparison t tests.
